# The Human Pathology Atlas for deciphering the prognostic features of human cancers

**DOI:** 10.1016/j.ebiom.2024.105495

**Published:** 2024-12-10

**Authors:** Meng Yuan, Cheng Zhang, Kalle Von Feilitzen, Martin Zwahlen, Mengnan Shi, Xiangyu Li, Hong Yang, Xiya Song, Hasan Turkez, Mathias Uhlén, Adil Mardinoglu

**Affiliations:** a Science for Life Laboratory, KTH-Royal Institute of Technology, Stockholm SE-17165, Sweden; b Guangzhou National Laboratory, Guangzhou, Guangdong Province 510005, China; c Department of Medical Biology, Faculty of Medicine, Atatürk University, Erzurum, Turkey; d Centre for Host-Microbiome Interactions, Faculty of Dentistry, Oral & Craniofacial Sciences, King’s College London, London SE1 9RT, UK

**Keywords:** Cancer, Transcriptomics, Survival, Systems biology

## Abstract

**Background:**

Cancer is one of the leading causes of mortality worldwide, highlighting the urgent need for a deeper molecular understanding and the development of personalized treatments. The present study aims to establish a solid association between gene expression and patient survival outcomes to enhance the utility of the Human Pathology Atlas for cancer research.

**Methods:**

In this updated analysis, we examined the expression profiles of 6918 patients across 21 cancer types. We integrated data from 10 independent cancer cohorts, creating a cross-validated, reliable collection of prognostic genes. We applied systems biology approach to identify the association between gene expression profiles and patient survival outcomes. We further constructed prognostic regulatory networks for kidney renal clear cell carcinoma (KIRC) and liver hepatocellular carcinoma (LIHC), which elucidate the molecular underpinnings associated with patient survival in these cancers.

**Findings:**

We observed that gene expression during the transition from normal to tumorous tissue exhibited diverse shifting patterns in their original tissue locations. Significant correlations between gene expression and patient survival outcomes were identified in KIRC and LIHC among the major cancer types. Additionally, the prognostic regulatory network established for these two cancers showed the indicative capabilities of the Human Pathology Atlas and provides actionable insights for cancer research.

**Interpretation:**

The updated Human Pathology Atlas provides a significant foundation for precision oncology and the formulation of personalized treatment strategies. These findings deepen our understanding of cancer biology and have the potential to advance targeted therapeutic approaches in clinical practice.

**Funding:**

The 10.13039/501100004063Knut and Alice Wallenberg Foundation (72110), the 10.13039/501100004543China Scholarship Council (Grant No. 202006940003).

**Research in context**

**Evidence before this study**

Since its establishment in 2017, the Human Pathology Atlas has been instrumental in linking gene expression profiling with patient survival outcomes, providing system-level insights and experimental validation across a wide range of cancer research. There is an urgent need for the systematic exploration of prognostic gene signatures to enhance the precision of cancer diagnostics and therapeutics.

**Added value of this study**

In this study, we annotated the pathological attributes of all protein-coding genes and established the correlations between gene expression and survival outcomes using global gene expression profiling. We observed significant variations in prognostic–gene associations across cancer types, and further investigated tumour heterogeneity and found that prognostic gene associations are highly specific to each cancer type.

**Implications of all the available evidence**

The Human Pathology Atlas offers a substantial basis for precision oncology. These discoveries would facilitate our comprehension of cancer biology and further provide insight into the progression of cancer treatment and precision medicine.

## Introduction

Cancer remains a significant global health challenge, with recent estimates indicating approximately 19.3 million new cases and almost 10 million deaths annually.^1^ In Europe, breast, colorectal, lung, and prostate cancers are the most frequently diagnosed cancers, collectively representing over half of all cases.^2^ Of particular concern is the high rate of premature mortality associated with cancer, imposing substantial societal and economic burdens.^3^ Extensive efforts have been invested in cancer research to develop effective treatment options and improve prognostic outcomes. However, universally effective and resilient treatments remain limited due to the heterogeneity of cancer.^4^, ^5^, ^6^, ^7^ This highlights the urgent need for a deeper understanding of the molecular mechanisms driving cancer pathogenesis and for the development of more effective, targeted and personalized treatment strategies. Cancer research has experienced significant evolution with advancements in computational power and the emergence of big data.^8^, ^9^, ^10^ Integrating multi-omics has propelled the field into a new era, where systems biology approaches can offer new insights into cancer’s complex pathology, bridging the existing gaps in our understanding of cancer pathogenesis and treatment efficacy.

Previously, we employed a systems biology approach to establish associations between gene expression profiles and patient survival outcomes, which we compiled into the Human Pathology Atlas.^11^ It is available in an open-access form as an essential component of the Human Protein Atlas (https://www.proteinatlas.org/), which has been integral to numerous cancer studies, furnishing experimental evidence and system-level insights to bolster research on biomarker identification and disease progression-related gene screening.^12^, ^13^, ^14^ Building upon the methodologies of our prior work, we have also identified tumour genes that correlate with patient survival, guiding us toward the discovery of promising drug targets and the development of inhibitory compounds capable of suppressing tumour cell growth and proliferation.^15^, ^16^, ^17^ These advancements emphasize the need for systematic exploration of prognostic gene signatures to enhance the precision of cancer diagnostics and therapeutics.

**Fig. 1:**
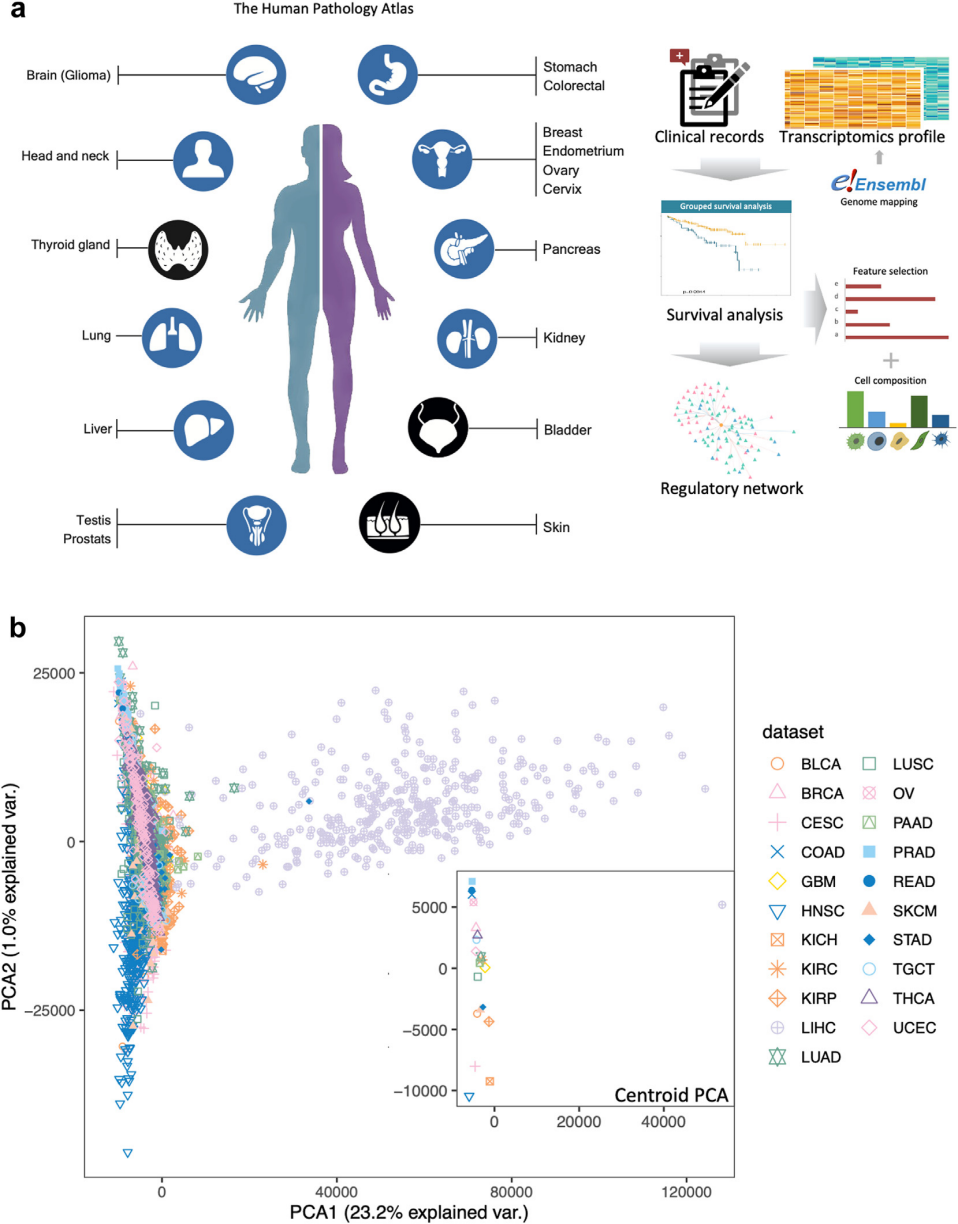
**Schematic overview.** (a) The overview of the workflow. The study covers 21 cancer types. Blue nodes indicate the cancer types have corresponding independent datasets of the same cancer type. (b) The PCA plot and centroid plot of 6918 patients among 21 cancer types. The plot is generated using mRNA expression levels, each axis reflects a principal component.

In this study, we re-annotated the pathological attributes of all protein-coding genes starting from the raw bam files and quantified gene expression as transcripts per million (TPM) to enable fair comparisons across a broad spectrum of genes and various cancer datasets. We also standardized gene expression on a quantile scale, allowing us to track shifts in gene expression from normal to tumour tissues. Furthermore, we updated the correlations between gene expression and survival outcomes using global gene expression profiling. Additionally, we compiled independent datasets from 10 different cancer types to identify a robust set of confidence prognostic genes (CPGs) that could enhance cancer research and potential clinical applications. Notably, we observed significant variations in prognostic–gene associations across cancer types. By focusing on liver hepatocellular carcinoma (LIHC) and colon adenocarcinoma (COAD), we investigated tumour heterogeneity and found that prognostic gene associations are highly specific to each cancer type. In the end, we constructed a prognostic regulatory network for kidney renal clear cell carcinoma (KIRC) and LIHC that incorporates these prognostic genes, paving the way for more comprehensive cancer investigations. The workflow of our study is depicted in Fig. 1a.

## Methods

### Pre-processing of data

We used the GDC client to download the raw BAM files of TPM for The Cancer Genome Atlas (TCGA) cohorts. After screening all samples across 21 cohorts, we retained data from 6918 donors who had both primary tumour solid tissue samples and associated clinical information. This clinical information was sourced from the TCGA Pan-Cancer Clinical Data Resource (TCGA-CDR)^18^ by categorizing the data according to cancer types.

We retrieved the global gene expression profiles (measured in Fragments Per Kilobase of transcript per Million mapped reads, FPKM) and clinical information for 442 donors from the International Cancer Genome Consortium (ICGC) database (http://icgc.org/), which includes data on breast cancer (BRCA-KR), liver cancer (LIRI-JP), ovarian cancer (OV-AU), and pancreatic cancer (PACA-AU). In our study, we limited our dataset to samples that included primary tumour solid tissue samples and clinical information. To avoid ambiguity in the expression data for donors with multiple tumour samples, we followed the criteria: preference was given to the sample labelled ‘C01’, or in the absence of such a label, we selected samples that were ‘untreated’, ‘included in PCAWG’, or had a ‘higher percentage of cellularity’. All FPKM values were converted to TPM, focusing on protein-coding genes to ensure data consistency.

The metadata and raw RNA-sequencing data for colorectal cancer were acquired from individuals who had surgery at Uppsala University Hospital in Sweden.^19^ The colon adenocarcinoma (COAD-UCAN) cohort consists of data from 486 patients, and the rectum adenocarcinoma (READ-UCAN) cohort comprises data from 207 patients.

The raw bam files and clinical information for 58 patients with Glioblastoma (GBM-GSE121720) were retrieved from the Gene Expression Omnibus (GEO) database (https://www.ncbi.nlm.nih.gov/geo/) using the accession number GSE121720.^20^ Only patients diagnosed with “primary glioblastoma” were included in our analysis. Additionally, we retrieved the RNA-seq data of 100 patients diagnosed with clear cell renal cell carcinoma (JAP-KIRC) from a Japanese cohort^21^ from the European Genome-phenome Archive using the accession number EGAS00001000509.

The metadata and raw RNA-sequencing data for lung cancer were collected from patients who underwent surgical treatment between 2006 and 2010 at Uppsala University Hospital in Uppsala, Sweden. These data are available in the NCBI SRA database using the accession number SRP074349.^22^^,^^23^ The lung adenocarcinoma cohort (LUAD-UCAN) includes data from 105 patients, and the lung squamous cell carcinoma (LUSC-UCAN) cohort comprises data from 68 patients.

For all cohorts with available raw data in this study, we employed the BEDTools^24^ for converting BAM to FASTQ files, and the Kallisto^25^ for calculating the TPM values for each gene (annotated by GRCh38 and Ensemble 103). During the analysis, we focused on the protein-coding genes, considering the mRNA expression value of the gene as the cumulative total of TPMs for all its transcripts. We included genes that exhibited an average expression level >1 across patients within each cancer type in our analysis. Furthermore, we included only patients with a recorded survival time of more than 0 days to minimize potential inaccuracies in clinical information. We conducted a Principal Component Analysis (PCA) to illustrate the overarching gene expression patterns across 21 different cancers. We finally clustered the cancers based on the mean expression level of genes, utilizing Euclidean distance as the metric for clustering.

### Ethics

Ethical approval was not required as only publicly available RNA-seq data was used.

### Classification of genes in cancers and normal tissues

The TPM values for normal tissues were acquired from the Human Protein Atlas.^26^ To ensure a fair comparison, we included only tissues with matched cancer types from the same sites. We categorized the protein-coding genes into five distinct groups according to their expression patterns in tumours and normal tissues, separately. The classifications are as follows: 1) Cancer/tissue enriched genes, which expression levels are at least four-fold higher in one cancer type/tissue compared with any others; 2) Group enriched genes, which expression levels are at least four-fold higher in a small number of cancer types or tissues (2–7 for cancer types, 2–6 for tissues); 3) Cancer/tissue enhanced genes, which expression levels are at least four-fold higher in one cancer type/tissue compared with the average expression level of that gene across all cancer types/tissues; 4) Low cancer/tissue specificity genes, which are expressed (TPM ≥ 1) in at least one cancer type/tissue, but not elevated in any of them; 5) Not detected genes, which expression levels are lower than 1 (TPM < 1) in all cancer types and tissues. A gene is denoted as an elevated gene if it’s classified as a cancer/tissue enriched, group enriched, or cancer-enhanced gene.

### Statistics

The Kaplan–Meier (KM) analysis was used to evaluate the association of gene expression with patients’ overall survival. We categorized each gene into two groups based on their TPM values for KM survival analysis and compared the survival outcomes using log-rank tests. To identify the optimal expression cut-offs for grouping, we examined all TPM values of each gene from the 20th to the 80th percentiles to stratify the patients. We examined significant differences in the survival outcomes of these groups and chose the cut-offs that yielded the lowest log-rank p value. The “survival” R package (version 3.5.5) was used for the Kaplan–Meier survival analysis, and “ggplot2” (version 3.5.0) was employed for visualizations. Genes were designated as prognostic genes (PGs) if they had log-rank p values less than 0.001. Additionally, a prognostic gene was considered unfavourable if the group with high expression had a higher number of observed events than expected; conversely, it was considered favourable if the number was lower. All analyses were executed using RStudio with R version 4.2.3.

The t-test was performed to compare the clinical information of different patient cohorts. The Wilcoxon rank sum test was applied to compare the mean values of cell ratio among different groups. The Kolmogorov–Smirnov test was used to assess the differences in pathway activity between patients who were alive and those who were deceased. Pathways with a p-value < 0.05 were extracted for downstream analysis.

### Correlation analysis

A gene qualifies as an overlapping prognostic gene across different datasets if it is identified as a prognostic gene in any dataset and shows a consistent directional effect (either consistently positive or consistently negative across all datasets). To evaluate the correlation between gene expression patterns across two different cohorts, we used the Spearman coefficient and the Jaccard Coefficient (JC). Furthermore, we employed the hypergeometric test to determine the statistical significance of the overlap between two gene lists. We performed the entire analytical process using RStudio with R version 4.2.3.

### Clinical feature ranking

We analysed the significance of clinical features using the Boruta SHAP algorithm,^27^ which integrates Boruta’s variable selection method with Shapley values, employing random forests to methodically determine variable importance. The Boruta algorithm iteratively identifies important features by comparing them against shadow features, which are randomly permuted versions of the original features. To determine the expression features, we applied the PCA to extract primary expression patterns, with a focus on the three most impactful principal components. We further transformed categorical clinical features, including cancer stage, race and sex, into numerical data using one-hot encoding. To achieve unbiased feature selection, we standardized all variables to a scale ranging from −1 to 1. To ensure the robustness of our feature selection method, we subjected all features to 100 shuffling iterations to bring them closer to a state of randomness. This entire analysis was carried out using Python.

### Prediction of cell-type proportion

We performed the analysis to identify cell types and their proportions within bulk RNA-seq datasets using the Dampened Weighted Least Squares (DWLS) approach.^28^ This technique is tailored to accurately deduce cell-type compositions, adjusting for any bias towards cells with either high gene expression levels or prevalence. Necessary reference profiles were sourced from single-cell RNA-seq data; for colorectal cancer, this data was retrieved from the GEO database using the accession number GSE178341. For hepatocellular carcinoma, the single-cell RNA-seq data was similarly obtained from the GEO database, linked to the accession number GSE149614.

### Construction of the regulatory networks for prognostic genes

We retrieved the KEGG^29^ pathway database from the Molecular Signatures Database (MSigDB).^30^ Quantitative assessment of molecular pathways and gene activity levels in tumour samples was performed to establish their associative patterns through the following steps: 1) The normalized enrichment score for each pathway was calculated for individual samples using a single sample-based Gene Set Enrichment Analysis (ssGSEA),^31^ and these scores were compiled into a pathway activity vector. Similarly, the VIPER algorithm^32^ was used to determine the activity score of transcriptional regulators (TRs) based on the ARACNe-inferred cancer network.^33^ These scores formed the basis for the TRs activity score vector. 2) Linear regression analysis was used to identify the regulatory relationship between gene activity (as the predictor) and pathway activity (as the response), denoted as the ‘slope’. A positive slope indicates a direct association, whereas a negative slope indicates an inverse relationship. 3) The robustness of these associations was validated through bootstrapping, performing 100 iterations to ensure statistical reliability. 4) Pathways that showed significant concordance with prognostic genes (PGs) were categorized as prognostic pathways, highlighting their potential influence on patient outcomes. The analyses were performed using the TR2PATH^34^ package (version 0.2.9) within RStudio. We applied the Kolmogorov–Smirnov test to assess the differences in activity between patients who were alive and those who were deceased. Pathways with a p-value > 0.05 were excluded from the analysis, which was performed using R.

### Role of funders

This study was funded by the Knut and Alice Wallenberg Foundation. The funder has no role in the study design, data collection, analysis, interpretation and writing of the report. The corresponding author had full access to all the data in this study and held the final responsibility for the decision to submit it for publication.

## Results

### Classification of genes in cancers and normal tissues

The RNA-seq data and corresponding clinical information for 6918 cancer patients diagnosed with 21 distinct human cancer types, as catalogued in TCGA (Supplementary Table S1), were downloaded. This dataset was uniformly processed through a consistent bioinformatics pipeline. Expression levels were subsequently normalized to TPM in order to enable comparative analysis across samples. We performed PCA to delineate the gene expression patterns among 21 different cancers (Fig. 1b). While a significant proportion of the cancers were closely aggregated, LIHC demonstrated pronounced heterogeneity in comparison to the other cancer types.

In this study, we adopted a comparable approach to categorize 19,652 protein-coding genes into five distinct categories based on their expression levels across various cancer types (Supplementary Fig. S1a) as previously described.^26^ Our analysis showed that a substantial portion (53.6%) of protein-coding genes were expressed in all cancers analysed, while an additional 12.1% of genes were not detected in any of the cancer types examined. The commonly expressed protein-coding genes were found to be enriched in typical cancer-related processes such as mRNA processing and cell cycle-related biological functions (Supplementary Fig. S1b). This enrichment aligns with the rapid cellular proliferation that occurs during tumourigenesis.

**Fig. 2:**
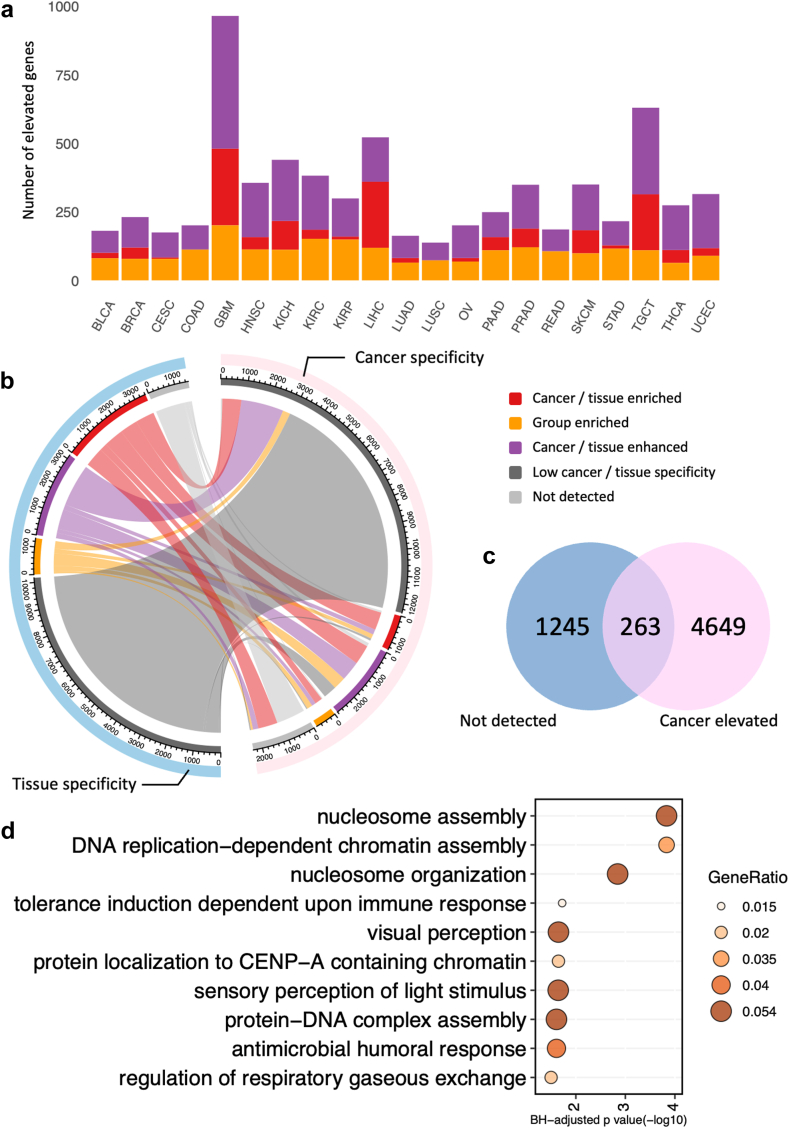
**Gene specificity classification.** (a) Number of elevated genes across 21 cancer types. A gene is considered elevated if classified as cancer/tissue-enriched, group-enriched, or cancer-enhanced. (b) Chord diagram showing the gene specificity shift pattern between 19,564 tissue- and 19,652 cancer-specificity genes based on TPM expression profiles. The outermost arc represents the tissue type (Blue - tissue specificity genes and pink – cancer specificity genes), while the second layer arcs indicate the specific gene specificity classification (Red – cancer/tissue enriched, yellow – group enriched, purple – cancer/tissue enhanced, grey – low cancer/tissue specificity and light grey – not detected). The axis of the second arc represents the gene numbers for each classification. (c) Venn diagram showing the 263 genes potentially shifting from low tissue specificity to cancer elevated status. (d) The cancer elevated genes are enriched in cell cycle-related progression.

Our analysis extended to the prevalence of genes with elevated expression levels across all cancer types, encompassing categories of cancer-enriched, group-enriched, and cancer-enhanced genes (Fig. 2a). Remarkably, glioblastoma multiforme (GBM), testicular germ cell tumours (TGCT), and LIHC exhibited the highest number of upregulated genes. This observation may be partly explained by the intrinsic heterogeneity of the brain, testis, and liver tissues, indicating that the elevated gene expression could be inherently connected to the properties of the tissues from which these cancers originate.

We downloaded the TPM expression profiles of genes in normal tissues from the Human Protein Atlas.^26^ Consequently, we sourced TPM profiles for 19 tissues corresponding to 17 distinct cancer types to delineate the gene expression patterns (detailed corresponding relationships can be found in Supplementary Table S2). Furthermore, we organized the 19,564 protein-coding genes sequenced in normal tissues into five categories across these tissue types (Supplementary Fig. S1c and d). In contrast to cancer states, a smaller fraction of genes (43.2%) was expressed across all normal tissue types, and a lower number of genes (7.7%) remain undetected in normal tissues. This pattern suggests a shift in gene expression from normal to cancerous tissues. To delve deeper into this phenomenon, we conducted a comparative analysis of gene specificity categories between normal and cancerous tissues (Fig. 2b).

We observed that the majority of genes with low tissue specificity maintained this characteristic during the transition from normal to tumour conditions. These genes are predominantly involved in essential cellular biological processes such as ribosome biogenesis and mitochondrial gene expression (Supplementary Fig. S1e). Additionally, genes that were categorized as having elevated expression in normal tissues exhibited a shift to various specificity categories in the context of cancer, reflecting the heterogeneity of gene expression across different cancer types. We particularly focused on genes that were not detected in normal tissues but showed elevated expression in cancerous conditions (Fig. 2c), as these genes may contribute to the progression of tumorigenesis. Among these, we identified 263 genes (Supplementary Table S3) predominantly involved in nucleosome assembly or DNA packaging processes (Fig. 2d), aligning with the rapid cellular proliferation typical of tumour progression.

### The identification of prognostic genes for cancers

**Fig. 3:**
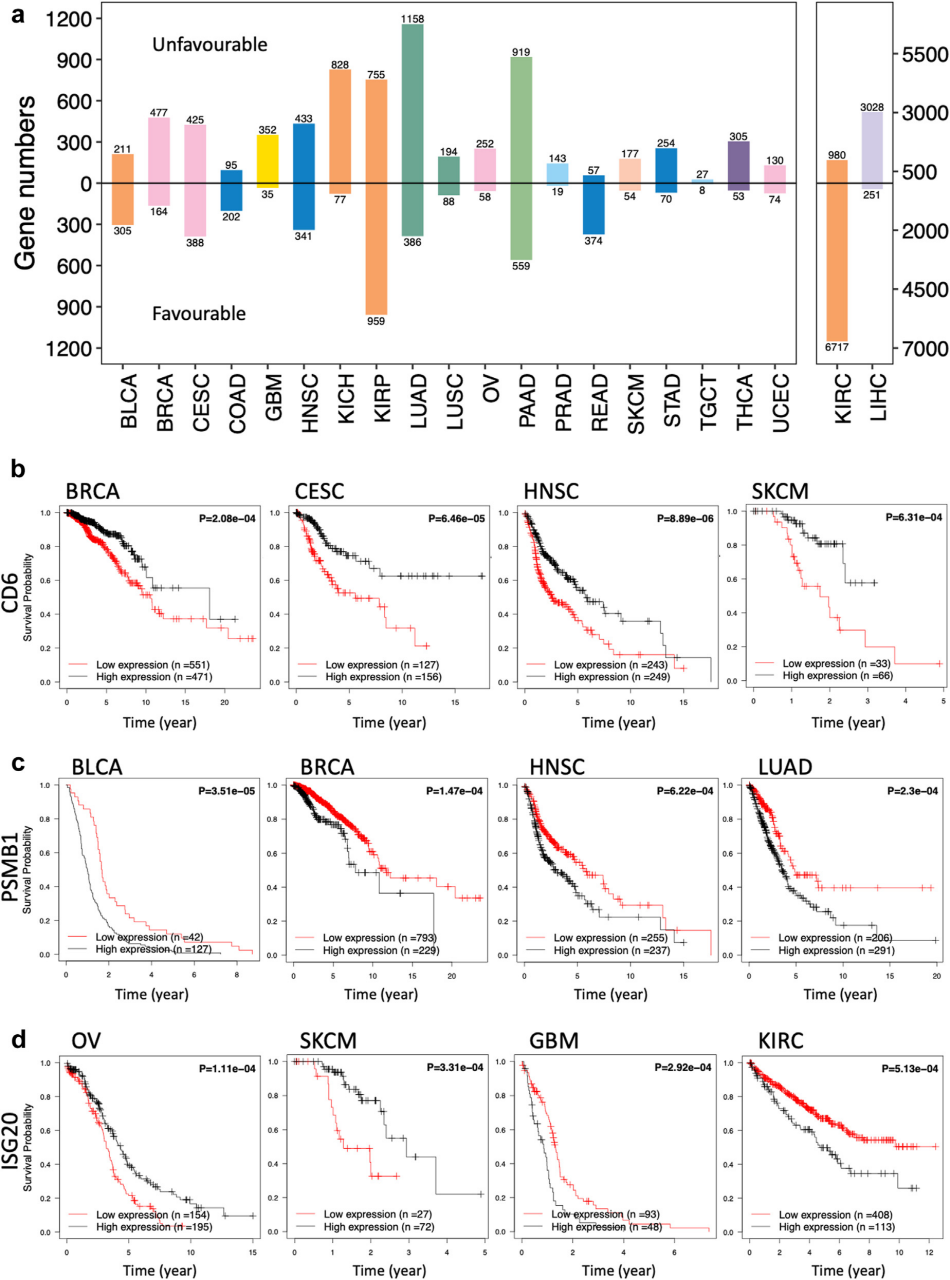
**Prognostic association between gene expression and survival outcome.** (a) The bar plot shows the prognostic gene numbers across 21 cancer types. (b) Gene *CD6* exhibits an unfavourable association with multiple cancers. (c) Gene *PSMB1* exhibits a favourable association with multiple cancers. (d) Gene *ISG20* exhibits different prognostic implications depending on the cancer type.

The KM analysis was employed to assess the relationship between the patient’s tumour transcriptomic profiles and clinical survival outcomes, from the recruitment in the study to the occurrence of death. As described in the Methods section and our previous research,^11^ patients were stratified into groups based on the high or low expression levels of the genes. The association between survival outcomes and gene expression levels was evaluated using KM analysis for each gene individually. As results, genes were labelled as ‘favourable’ and ‘unfavourable’ if high expression correlated with better or poor survival outcomes, respectively. We analysed the number of PGs for each cancer type (Fig. 3a) and observed that KIRC and LIHC had the highest numbers of PGs. In KIRC, the majority of PGs (87.3%) were categorized as favourable genes, whereas the majority of PGs (92.3%) were categorized as unfavourable genes in LIHC.

The prognostic significance of the genes varied across cancer types, with some demonstrating consistent prognostic values. For example, *CD6*, a crucial gene for T-cell activation, is identified as a favourable prognostic marker in multiple cancers, such as breast invasive carcinoma (BRCA), cervical squamous cell carcinoma and endocervical adenocarcinoma (CESC), head and neck squamous cell carcinoma (HNSC), and skin cutaneous melanoma (SKCM), as shown in Fig. 3b. Similarly, *PSMB1*, the non-catalytic component of the proteasome complex, was implicated in poor survival outcomes across several cancer types (Fig. 3c), namely bladder urothelial carcinoma (BLCA), BRCA, HNSC and lung adenocarcinoma (LUAD). Our findings suggest a potential underlying commonality in the regulatory mechanisms across these cancers.

Conversely, certain genes exhibited distinctly different prognostic significance depending on the cancer type. As shown in Fig. 3d, interferon-induced anti-viral exoribonuclease (*ISG20*), acting on single stranded RNA and involved in immune and inflammatory responses, correlated with improved survival in ovarian serous cystadenocarcinoma (OV) and SKCM. However, its high expression is indicative of poorer survival in GBM and KIRC. These findings align with previously published research about these cancers.^35^, ^36^, ^37^

### Validation of prognostic genes in different cancer cohorts

**Fig. 4:**
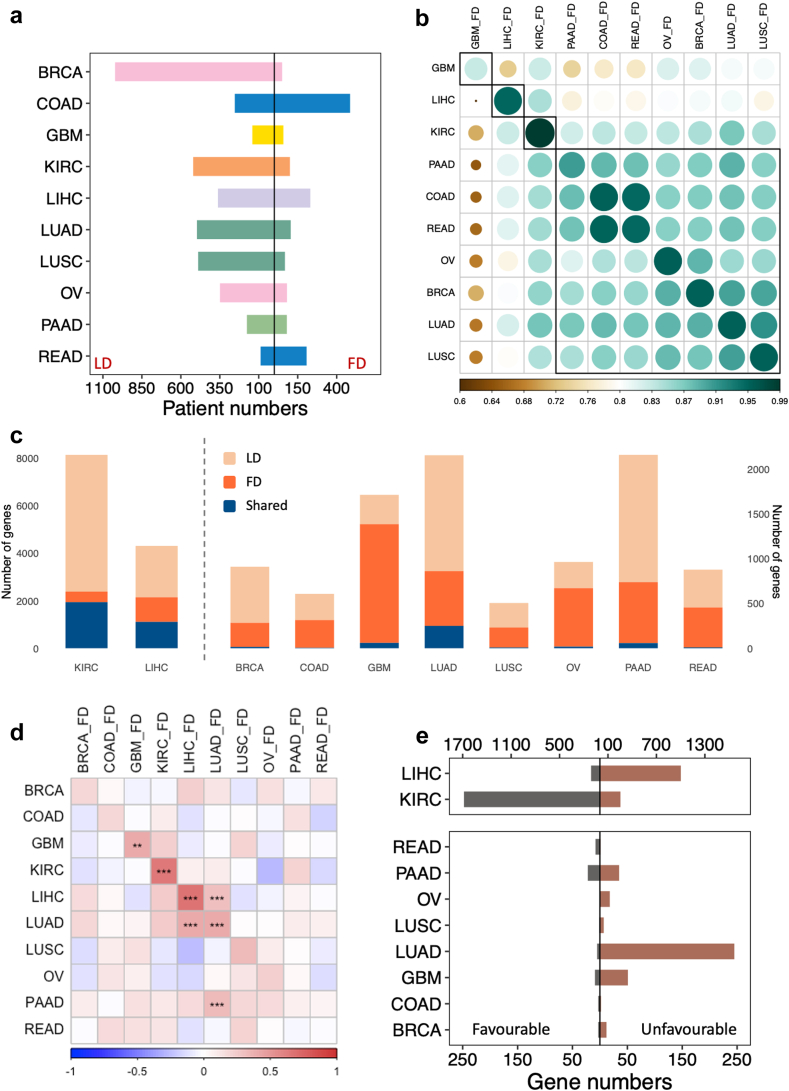
**The confidence prognostic genes.** (a) Patients number in the leading datasets (LD) cohorts and follow-up datasets (FD) across 10 cancer types. (b) Mean expression level correlations in 10 cancer types between LD and FD. The circle size and colour represent the value of the Spearman correlation. A smaller size indicates a lower correlation (min is LIHC-FD & GBM-FD, max is KIRC & KIRC-FD). (c) The stack plot shows the number of prognostic genes in 10 cancer types. (d) The Spearman correlation of KM coefficient of prognostic genes among cancer types. The hypergeometric test was applied to examine the overlap significance, p values are denoted as: ∗p < 0.05, ∗∗p < 0.01, and ∗∗∗p < 0.001. (e) Favourable and unfavourable gene numbers in shared prognostic genes.

In the previous version of the Human Pathology Atlas,^11^ we reported significant variability in the number of PGs across different cancer types. To reduce dependence on a single dataset, we compiled 10 follow-up datasets (FDs) from various sources, each corresponding to one of the cancer types included in the leading datasets (LDs), specifically the TCGA cohorts (refer to Fig. 4a and Supplementary Table S4). These FDs were re-annotated using the same bioinformatics pipeline and reference genomes, and a consistent approach was applied to filter their clinical records.

We analysed the connectivity among LDs and FDs for the 10 cancer types using PCA based on the expression patterns of all protein-coding genes (Supplementary Fig. S2a). Notably, the LIHC-FD exhibited distinct expression patterns that aligned closely with those of the LD cohort. The centroid plot in the upper right corner further demonstrated that LD and FD share similar variances in PCA. Additionally, a clearer clustering trend was observed in the dendrogram plot (Supplementary Fig. S2b), showing that dataset pairs of the same cancer type generally clustered more closely. However, a significant divergence was noted in the breast cancer (BRCA) FD, which could be attributed to its specific age demographic—comprising solely young individuals aged 25 to 35 years—differing from the broader age range (25–90 years old) in the LD.

The cluster heatmap supports our observations (Fig. 4b), aligning with the findings discussed above. Although the Spearman correlation coefficients for all LDs and FDs were generally above 0.6, indicating a robust association, expression profiles were most conserved within the same cancer type—reflecting underlying biological consistencies. Notably, GBM emerged as the most distinct cancer type, with the Spearman correlation between GBM datasets being the lowest among all dataset pairs of the same cancer type. In contrast, KIRC and LIHC displayed the highest Spearman correlation between their respective LD and FD. Consistent with the PCA results, LIHC also showed a considerably low Spearman correlation with datasets of other cancer types. Aside from these three cancers, other cancer types clustered together, suggesting greater homogeneity in gene expression patterns. Within this larger cluster, COAD and rectum adenocarcinoma (READ), both originating from the same organ and often collectively referred to as colorectal cancer, showed higher intragroup similarity.

To assess the robustness of the PGs, we performed KM analysis in the 10 FDs using the same pipeline. A gene was considered a confidence gene if it consistently demonstrated prognostic value across both the LD and FD of the same cancer type. Consequently, we were able to identify shared CPG for the two independent data sets (Fig. 4c). KIRC and LIHC exhibited the highest number of shared prognostic genes, while cancers such as COAD and lung squamous cell carcinoma (LUSC) were found to have fewer prognostic genes identified by the two independent cohorts.

We further assessed the repeatability of PG identification by calculating the Spearman correlation for KM coefficients of genes between the LDs and FDs. As shown in Fig. 4d, there was a general trend of positive correlation between the KM coefficients for the same cancer type. The PGs from the LDs and FDs for four cancers (GBM, KIRC, LIHC, and LUAD) showed significant overlap (hypergeometric test, p < 0.05), indicating a robust expression–survival association for these cancers. Notably, KIRC and LIHC displayed the highest correlation coefficients (r = 0.64, JC = 0.26 for KIRC; r = 0.66, JC = 0.24 for LIHC, Fig. 4e). We observed that most of the identified KIRC CPGs were favourable and these genes are associated with the regulation of cell cycle-related transcription, whereas the majority of LIHC CPGs were unfavourable and they are enriched in biogenesis, RNA assembly and gene expression.

### Cell proportions in cancer have a major effect on prognostic genes

Significant differences were observed in the CPGs across the 10 cancer types studied. Given that LDs and FDs originated from various sources, it was not possible to account for all variables in our analysis. To interpret these results from a systems biology perspective, we focused on LIHC, which showed high consistency in CPGs, and COAD, which displayed low consistency, for more detailed analysis.

**Fig. 5:**
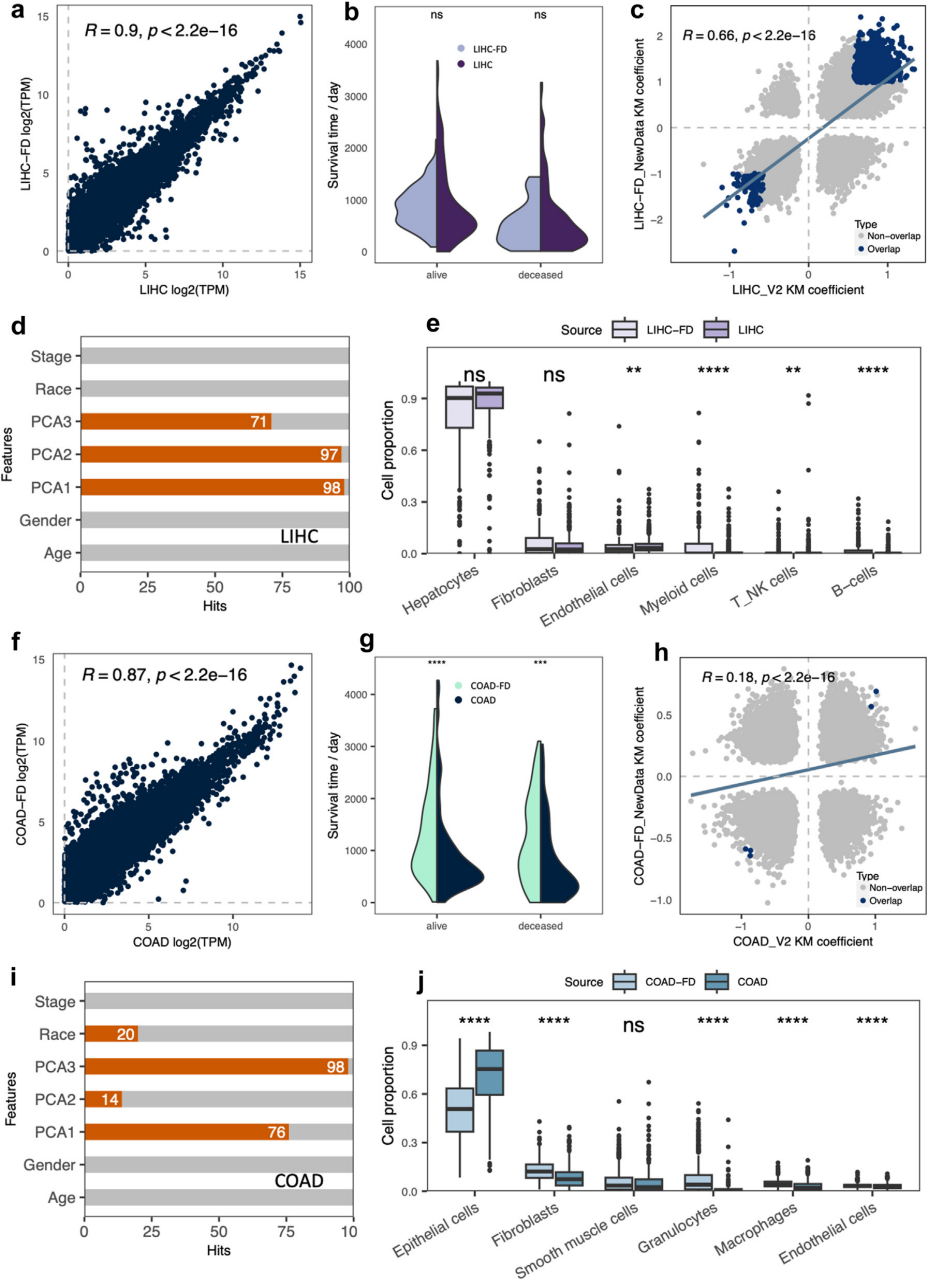
**Features that affect prognostic estimation.** (a) The spearman correlation of gene average expression level between LIHC and LIHC-FD cohorts. (b) The violin plot shows that patient survival days distribution between LIHC and LIHC-FD do not have statistical differences (p = 0.6 for alive group and p = 0.21 for deceased group, estimated by t-test). (c) The confidence prognostic genes were estimated by KM analysis. (d) Feature contribution to survival outcome in LIHC (by Bruta Sharp, iteration times = 100). (e) The cell proportion of major cell types has no statistical differences between LIHC and LIHC-FD cohorts, with hepatocytes p = 0.1, Fibroblasts p = 0.87 (estimated by Wilcoxon rank sum test). (f) The spearman correlation of gene average expression level between COAD and COAD-FD cohorts. (g) The violin plot shows that patient survival days distribution in COAD and COAD-FD have statistical differences (p = 8e-08 for alive group and p = 0.00035 for deceased group, estimated by t-test). (h) The confidence prognostic genes were estimated by KM analysis. (i) Feature contribution to survival outcome in COAD (by Bruta Sharp, iteration times = 100). (j) The cell proportion of major cell types (top 6) has statistical differences between COAD and COAD-FD cohorts (estimated by Wilcoxon rank sum test). The p values are denoted as: ∗p < 0.05, ∗∗p < 0.01, ∗∗∗p < 0.001 and ∗∗∗∗p < 0.0001.

The LIHC datasets exhibited a strong correlation in expression profiles across all protein-coding genes (Fig. 5a) and negligible differences in survival times (Fig. 5b), along with a considerable number of shared PGs. As previously mentioned, LIHC showed the highest similarity in KM coefficients (Fig. 5c). Using the Boruta SHAP algorithm, which identifying the most relevant features to outcomes, we evaluated critical features influencing patient survival. Four well-documented clinical variables (cancer stage, race, sex, and age) and the top three principal components of the LD expression profiles, representing overall expression patterns, were assessed. Across 100 iterations, expression principal components consistently emerged as significant factors for survival, while other clinical attributes were not emphasized (Fig. 5d). Furthermore, both LIHC datasets displayed high congruence in cell-type proportions, with hepatocytes constituting the majority (>90%, Fig. 5e), indicating high cellular homogeneity within the samples.

In contrast to LIHC, COAD displayed distinct characteristics in all evaluated aspects. As previously noted, the gene expression profiles across all protein-coding genes of COAD were less distinctive compared to those of LIHC (Fig. 5f). Significant differences were also observed in the survival times between the living and deceased patient groups (Fig. 5g), suggesting that the COAD cohorts might be subject to highly divergent exposure factors. These multiple discrepancies likely contributed to the lower confidence in PGs identified for COAD (Fig. 5h).

In our survival analysis (Fig. 5i), expression principal components for COAD were identified as important less frequently compared to LIHC, whereas ‘Race’ was more frequently recognized as a significant factor (N = 20), even surpassing PCA2. Additionally, the major cell types within COAD, namely epithelial cells and fibroblasts, showed significantly different proportions across the datasets, yet both were present in low percentages (Fig. 5j). These comparisons underscore the intrinsic differences between the two COAD cohorts, both in terms of the clinical characteristics of the patients and the cellular composition of the sequenced samples.

The KM analysis assigns greater weight to the survival days of deceased patients because they represent “completed event records.” When comparing survival days between cohort pairs (Supplementary Fig. S3 and Table S5), it was found that, of the ten cancer dataset pairs, six showed no statistical difference in the survival days of the deceased patient group. Among these, cancer types such as KIRC, LIHC and LUAD demonstrated significant consistency between the LD and FD. Although there was no statistical difference in survival among deceased patients with BRCA, intrinsic biological differences are evident; the FD for BRCA includes younger patients (ages 25–35) compared to the broader age range (26–90 years) in the LD, which could influence the overlap of prognostic genes. Additionally, variations in cancer subtypes or treatment modalities can lead to notable deviations between datasets. For instance, in the READ cohort, 43.2% of patients underwent pharmaceutical therapy and 56.8% received radiation therapy. In contrast, the majority of the (READ-FD) cohort did not receive any treatment (77.7%), with only a small fraction undergoing pharmaceutical intervention. This divergence in treatment approaches is reflected in the substantial variation in survival durations observed between the living and deceased patients across both cohorts.

### Construction of the cancer regulatory networks for prognostic genes

Our study revealed that KIRC and LIHC are characterized by a strong correlation between gene expression profiles and prognostic outcomes. Despite the abundance of PGs, selecting the most efficacious genes for treatment remains challenging. To improve the specificity of PGs selection, we construct a regulatory network for KIRC prognostic genes. This network serves as a strategic framework to guide the selection of genes within relevant pathways, potentially streamlining the identification of therapeutic targets (see Methods section for a detailed methodology).

**Fig. 6:**
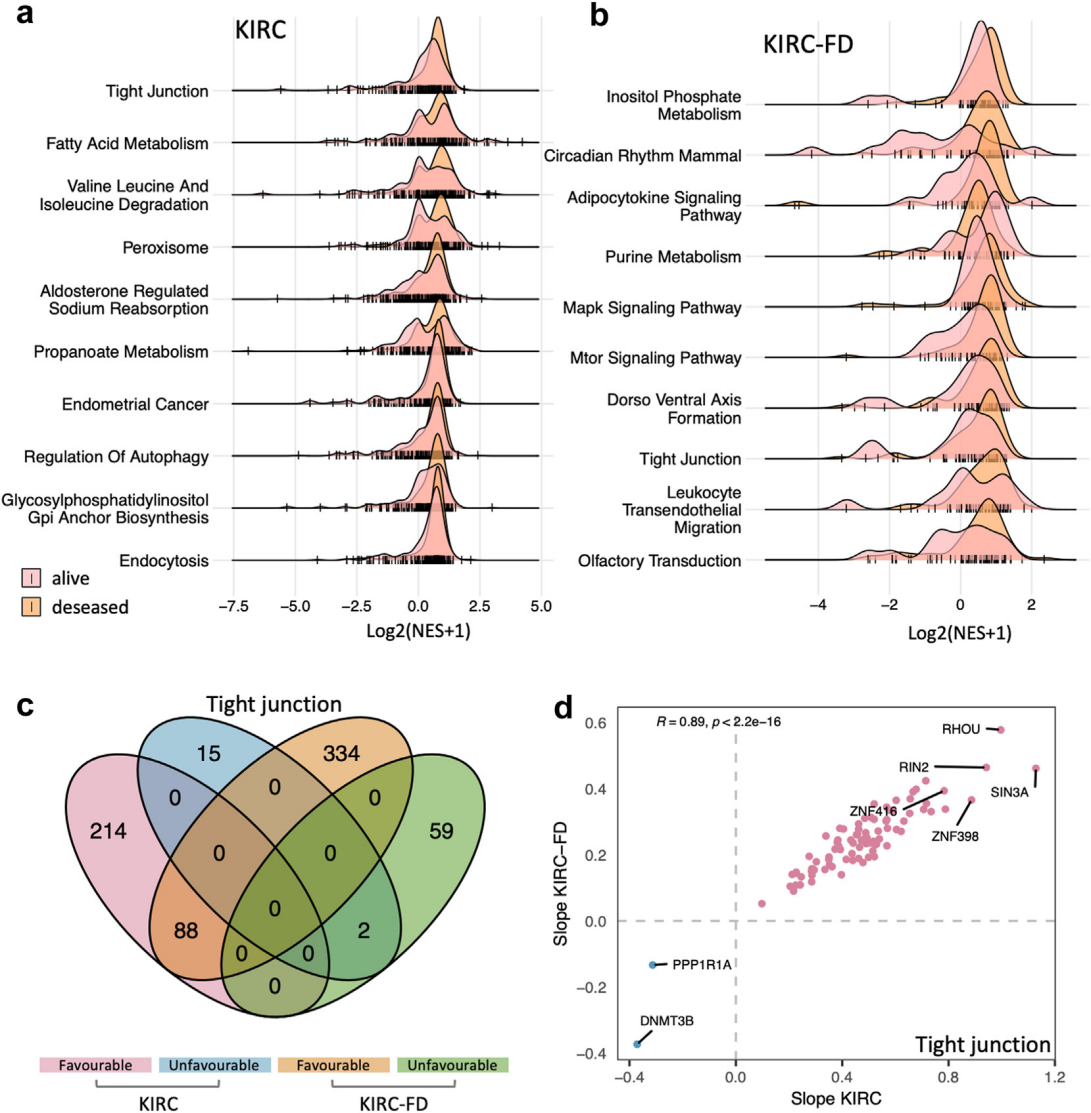
**Prognostic pathway regulatory network of KIRC.** (a) The pathways that differently activated among alive and deceased patients in KIRC. (b) The pathways that are differently activated among alive and deceased patients in KIRC-FD. (c) The TRs that regulate tight junction pathways are significantly associated with the patient prognosis of KIRC and KIRC-FD. (d) The CPGs deprived of (c) and KM analysis showed high consistency of activity in KIRC and KIRC-FD.

We downloaded a comprehensive set of 186 KEGG pathways with their associated genes from MSigDB.^30^ For each sample in our dataset, we calculated the activity score for each of these pathways. The top 10 pathways with the lowest p values (p < 0.05, by Kolmogorov–Smirnov test) that significantly have different activity scores among the alive and deceased patient groups in KIRC-LD were shown in Fig. 6a, while the top pathways in KIRC-FD were shown in Fig. 6b. The tight junction pathway, which emerged as the shared top pathway of different activity in both LD and FD cohorts, was thus regarded as a potential key pathway related to different survival outcomes. It plays a key role in cell adhesion and permeability in epithelial cells and shows reduced activity in KIRC samples compared to non-tumourous tissue.^38^ Additionally, it has been implicated in the progression of more advanced tumour pathology by contributing to cell proliferation, migration and differentiation.^39^^,^^40^

For KIRC, we utilized the ARACNe-inferred KIRC network,^33^ which includes 6054 transcriptional regulators (TRs) and their gene regulatory associations. We conducted a linear regression analysis to assess the correlation between pathway activities and the activities of major identified TRs, with the robustness of these correlations verified via bootstrap analysis (n = 100 iterations). In KIRC, we identified 529 TRs that exhibit regulatory interactions with the tight junction pathway (Supplementary Table S6). Of these, 319 TRs (60.03%) also demonstrated a correlation with patient survival outcomes in KIRC. In the KIRC-FD, 2051 TRs were implicated in the regulation of the tight junction pathway, with 23.55% (483 TRs) associated with patient survival in KIRC-FD.

Comparative analysis between the KIRC-LD and KIRC-FD cohorts revealed 90 TRs involved in the tight junction pathway, which were also concurrently identified as KIRC CPGs in previous KM analysis (Fig. 6c). These TRs exhibited a high correlation in slope value (r = 0.89, by Spearman Coefficient, Fig. 6d). The majority of these TRs were categorized as favourable CPG biomarkers (88 TRs), each showing positive regulation of the tight junction pathway. In contrast, two TRs, DNMT3B and PPP1R1A, were classified as unfavourable KIRC CPGs, displaying a negative regulatory relationship with the tight junction pathway. The impairment of this pathway may play a critical role in KIRC pathogenesis, aligning with our findings where the overexpression of the two unfavourable CPGs could decrease pathway activity, potentially accelerating tumour progression and resulting in worse patient survival outcomes. Moreover, the two TRs have been extensively investigated across multiple studies and recognized as potential candidates for cancer therapy,^41^^,^^42^ indicating their potential application in future KIRC research.

We applied a similar methodology to construct the regulatory network for LIHC prognostic genes (Supplementary Table S7). Differential activation of the purine metabolism and RNA polymerase pathways was observed between alive and deceased patients with LIHC, as well as LIHC-FD, as shown in Supplementary Fig. S4a and b. Within the regulatory framework of the purine metabolism pathway, 209 TRs were also identified as LIHC CPG (Supplementary Fig. S4c). The inhibition of purine metabolism is known to suppress the progression of hepatocellular carcinoma (HCC).^43^ Notably, genes classified as unfavourable exhibited a positive regulatory association with purine metabolism, suggesting a potential inhibition through the unfavourable genes.

In contrast, 165 TRs, which also align with CPGs, were identified concerning the RNA polymerase pathway, as shown in Supplementary Fig. S4d. Although the activity scores of CPGs within survival-differential pathways indicated a lower Spearman correlation in LIHC, we observed three genes—TAF15, CHEK1, and PDCD6—as having the highest slope values in both purine metabolism and RNA polymerase pathways (Supplementary Fig. S4e and f). These genes have been implicated in the inhibition of HCC progression^44^^,^^45^ and cellular migration,^46^ illustrating their potential as targets for the development of effective HCC treatment.

### The impact of updated datasets on prognostic genes

In the Human Pathology Atlas, our focus was on protein-coding genes, deriving expression levels from the aggregate of protein-coding transcripts. Utilizing Ensembl release 103, which includes updated gene or transcript classification for over 3000 genes, updating our gene classification was a crucial initial step in our analysis. We performed a correlation analysis of expression profiles to detect changes in overall expression patterns between the previous version^11^ and presented version. Despite employing different gene quantification methods (previously FPKM and currently TPM), the average Spearman correlation coefficient remained above 0.8, which is relatively low considering the samples are generally the same.

Our dataset has been updated with the latest clinical records from the TCGA database, meticulously comparing changes on a case-by-case basis. Significant updates include alterations in cohort sample sizes, with notable reductions observed across most cancer types (Supplementary Fig. S5a). For instance, the sample size for uterine corpus endometrial carcinoma (UCEC) decreased by 67.5% due to the unavailability of raw bam files for 365 patients, resulting in the lowest expression correlation (r = 0.84, by the Spearman coefficient). Adjustments in patients’ clinical information were also evident; for example, the survival days for a patient sample of BRCA was revised to 1468 days (2024 days less than the previous record), and the survival status of a patient with CESC was updated to deceased with no change in survival time.

We conducted a comparative analysis of PGs across two versions of the dataset, as shown in Supplementary Fig. S5b. The number of PGs for each cancer type is listed, along with their respective categories, with the significance of overlap indicated by asterisks. While high consistency was anticipated and observed within the same cancer types, the gene lists are not entirely identical. This discrepancy highlights the sensitivity of survival analysis to data variations, particularly changes in expression levels and clinical information.

## Discussion

In this study, we compiled and updated publicly available cancer datasets and conducted KM survival analyses to systematically explore the relationship between gene expression and patient survival outcomes. The identification of PGs holds significant biological relevance, as these genes provide insights into the prognostic implications of gene expression. Generally, genes involved in cell proliferation and mitosis correlate with unfavourable prognoses, while those related to cellular differentiation and immune activation typically signify more favourable outcomes. Understanding the function of these prognostic genes facilitates insights into the heterogeneous gene associations in cancers, thereby contributing to the discovery of cancer target genes and key biological processes relevant to precision medicine.

Our findings revealed distinct patterns across various cancer types. Notably, KIRC and LIHC demonstrated a significant number of PGs, indicating a robust correlation between gene expression profiles and survival outcomes in these cancers. These PGs were further validated using independent datasets. The high expression correlation observed in both the initial and follow-up datasets for KIRC and LIHC suggests better consistency in the disease pathology rather than significant variability among patients. This consistency was also supported by cell type analysis derived from the LIHC datasets, suggesting that LIHC may exhibit uniform behaviour across different studies, potentially due to the homogeneous nature of the tissue involved.

However, the impact of gene expression on cancer prognosis varies across different cancer types. The fundamental complexity of cancer, which includes genetic diversity, epigenetic modifications, comorbidities, environmental factors, and lifestyle choices, contributes differently to disease progression and patient survival.^47^, ^48^, ^49^, ^50^ Certain cancers, such as TGCT and Prostate Adenocarcinoma (PRAD), have been found to have a significantly smaller set of prognostic genes, suggesting a potentially weaker correlation between gene expression and survival outcomes in these cases.

Furthermore, our methodology for selecting PGs was stringent, utilizing a p-value threshold of less than 0.001 to ensure robust statistical significance. This rigorous cut-off minimizes the influence of potential gene expression fluctuations on our results. However, the unique characteristics of each tumour type may necessitate a more flexible approach to cut-off criteria, potentially adapting them to better match the specificities of individual cancers. Such a nuanced consideration of cut-off thresholds could facilitate a more tailored and insightful analysis when studying specific cancer types.^15^, ^16^, ^17^

Additionally, we constructed prognostic networks for KIRC and LIHC, showcasing how cancer-specific prognostic genes can be integrated into cancer research. These CPGs can serve as a systematic reference to streamline the selection of gene candidates and further identify those with strong associations with survival outcomes.

Our comparative analysis of clinical information across different cancer cohorts showed that even minor discrepancies can significantly affect survival analysis outcomes. This underscores the need for meticulous examination of the original data before conducting survival analysis to reduce the risk of error and ensure the reliability of the study’s conclusions. However, one limitation of this study is the need to expand the number of FDs, which would provide broader insights into additional cancer types and deepen our understanding of cancer. Furthermore, the clinical data of cancer patients is often noisy and lacks standardized protocols, which limits the availability of clinical factors for evaluating their impact on overall survival. While gene expression patterns serve as crucial biomarkers in some cancers, their prognostic value may be less pronounced in others, necessitating a comprehensive approach to understanding and predicting cancer survival. Future studies should strive to incorporate a broader range of data to enhance the accuracy of survival analyses and minimize the effects of inconsistent clinical information.

In conclusion, we employed the Kaplan–Meier analyses to determine the prognostic significance of protein-coding genes in patients’ survival across 21 cancer types. We curated lists of genes with favourable and unfavourable prognostic values. Additionally, we compiled a robust list of genes for 10 cancer types, confirming their prognostic value through validation with independent cancer cohorts. The comprehensive analysis and use of large datasets increases the statistical power and transparency of the study, enabling us to identify significant associations with confidence. Our analysis of clinical information indicated that gene expression patterns significantly impacted survival predictions, particularly in KIRC and LIHC cancer types. The results of this study are presented in the updated Cancer section of the open access Human Protein Atlas resource (www.proteinatlas.org).

## Contributors

M.Y. contributed to the data curation, investigation, methodology, visualization and writing-original draft in this study. C.Z. contributed to the data curation, investigation, methodology, project administration, supervision and writing-review and editing. K.F. contributed to the software development. M.Z. contributed to the software development. M.S. contributed to the data curation, methodology and writing-review and editing. X.L. contributed to the methodology and writing-review and editing. H.Y. contributed to the methodology and writing-review and editing. X.S. contributed to the methodology and writing-review and editing. H.T. contributed to the writing-review and editing. M.U. contributed to the project administration, supervision and writing-review and editing. A.M. contributed to the methodology, project administration, supervision, funding acquisition and writing-review and editing. C.Z and M.Y. accessed and verified the underlying data. A.M. is responsible for the decision to submit the manuscript. All the authors read and approved the final manuscript.

## Data sharing statement

All data needed to evaluate the conclusions in the paper are present in the paper and/or the Supplementary Materials. The scripts required to reproduce the results presented in this paper are available in the GitHub repository (https://github.com/cellur-m/pathology_atlas).

## Declaration of interests

AM and MU are the founders and shareholders of ScandiBio Therapeutics, ScandiEdge Therapeutics and Atlas Antibodies (MU). The other authors declare no competing interests.

## Appendix A Supplementary data

Supplementary Fig. S1 **Figure S1. Gene specificity classification among tissues.** (a) Number of each gene specificity classification in cancer tissues. (b) The enriched biological functions in protein-coding genes expressed in all cancer types. (c) The number of each gene specificity classification in normal tissues. (d) Number of elevated genes across 17 tissue types. (e) The enriched biological functions in low tissue specificity expressed genes in both normal and cancer tissues.

Supplementary Fig. S2 **Figure S2. The expression pattern of 10 cancer types.** (a) PCA plot of hallmark gene expression distribution of 8384 patients. The plot is generated using mRNA expression levels, each axis reflects a principal component. (b) The dendrogram of protein-coding genes shows the clustering results based on the expression features in 10 cancer types with corresponding FD.

Supplementary Fig. S3 **Figure S3. Clinical information overview**.

Supplementary Fig. S4 **Figure S4. Prognostic pathway regulatory network of LIHC.** (a) The pathways that differently activated among alive and deceased patients in LIHC. (b) The pathways that are differently activated among alive and deceased patients in LIHC-FD. (c) The TRs that regulate the purine metabolism pathway are significantly associated with the patient prognosis of LIHC and LIHC-FD. (d) The TRs that regulate the RNA polymerase pathway are significantly associated with the patient prognosis of LIHC and LIHC-FD. (e) The CPGs deprived of (c) and KM analysis showed high consistency of activity in LIHC and LIHC-FD. (f) The CPGs deprived from (d) and KM analysis showed high consistency of activity in LIHC and LIHC-FD.

Supplementary Fig. S5 **Figure S5. Comparison of two versions.** (a) The sample number comparison among 21 cancer types. (b) The Spearman correlation of KM coefficient of prognostic genes among cancer types. The hypergeometric test was applied to examine the overlap significance, p values are denoted as: ∗ p<0.05, ∗∗ p<0.01, and ∗∗∗ p<0.001.
