# Supplementary figures and images for: The Human Pathology Atlas for deciphering the prognostic features of human cancers
Source: eBioMedicine. 2024 Dec 10;111:105495. doi: 10.1016/j.ebiom.2024.105495 (PMC11683280; doi:10.1016/j.ebiom.2024.105495)

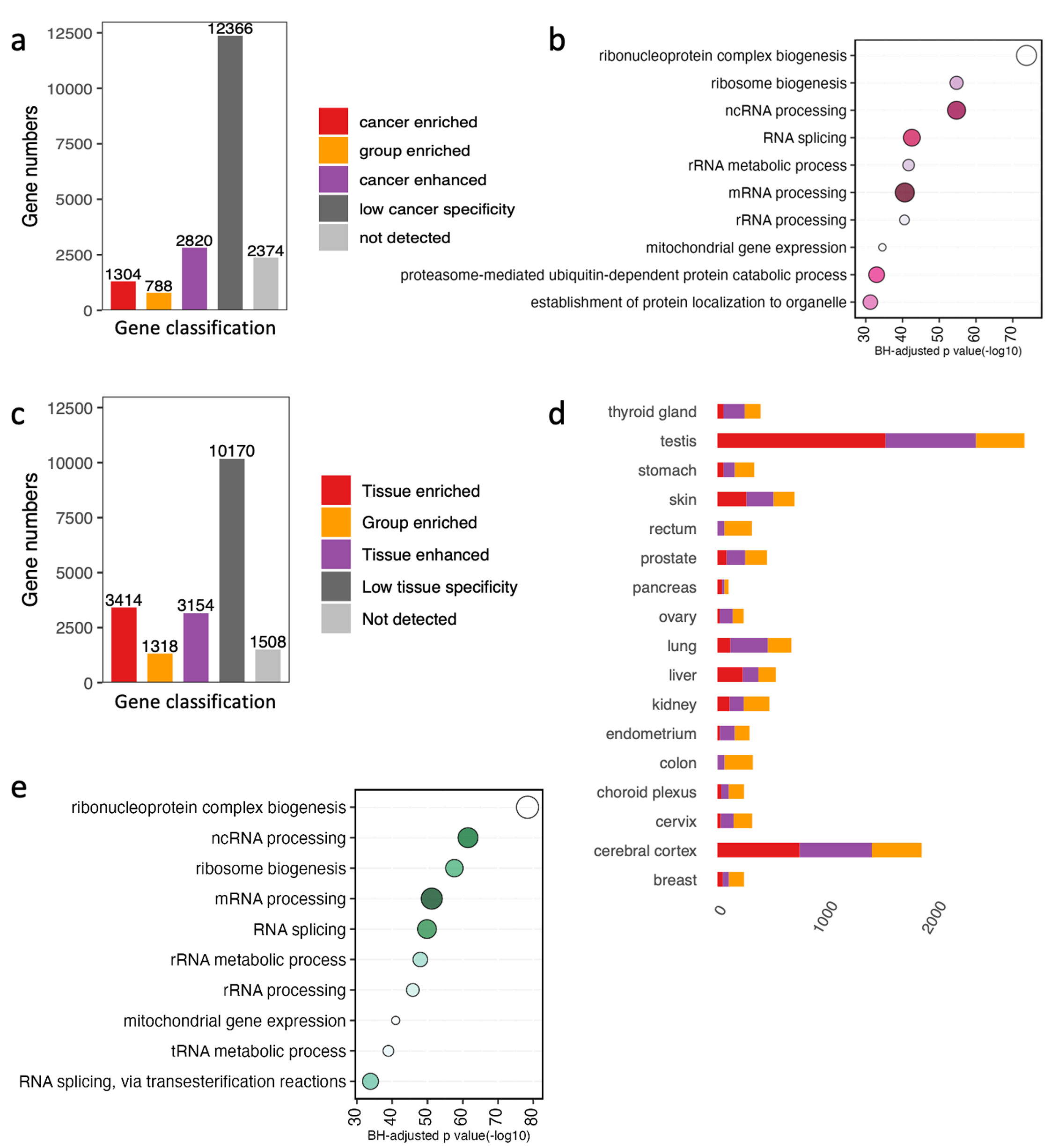

Supplement: Supplementary Fig. S1 — Figure S1. Gene specificity classification among tissues. (a) Number of each gene specificity classification in cancer tissues. (b) The enriched biological functions in protein-coding genes expressed in all cancer types. (c) The number of each gene specificity classification in normal tissues. (d) Number of elevated genes across 17 tissue types. (e) The enriched biological functions in low tissue specificity expressed genes in both normal and cancer tissues. [file figs1.jpg]

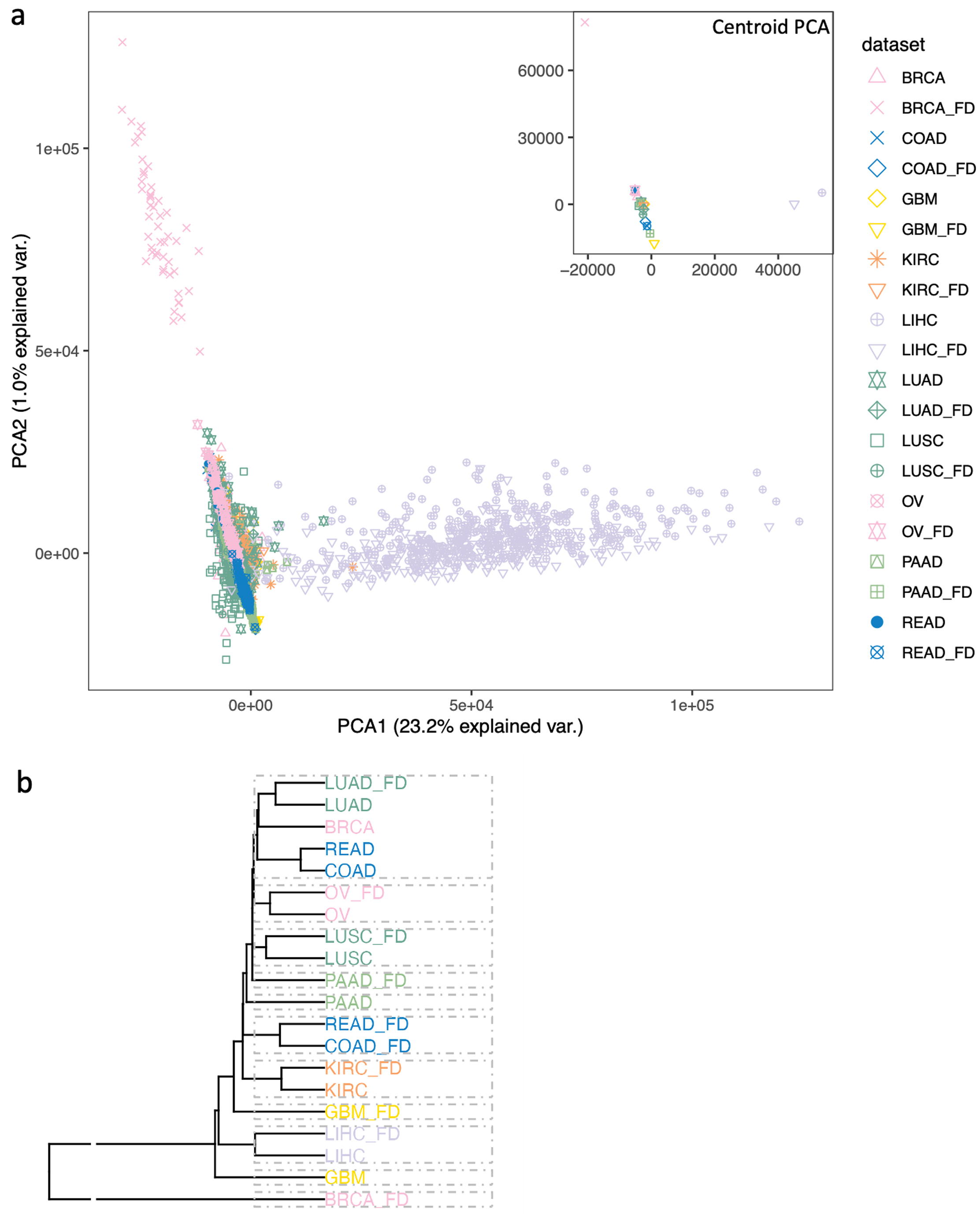

Supplement: Supplementary Fig. S2 — Figure S2. The expression pattern of 10 cancer types. (a) PCA plot of hallmark gene expression distribution of 8384 patients. The plot is generated using mRNA expression levels, each axis reflects a principal component. (b) The dendrogram of protein-coding genes shows the clustering results based on the expression features in 10 cancer types with corresponding FD. [file figs2.jpg]

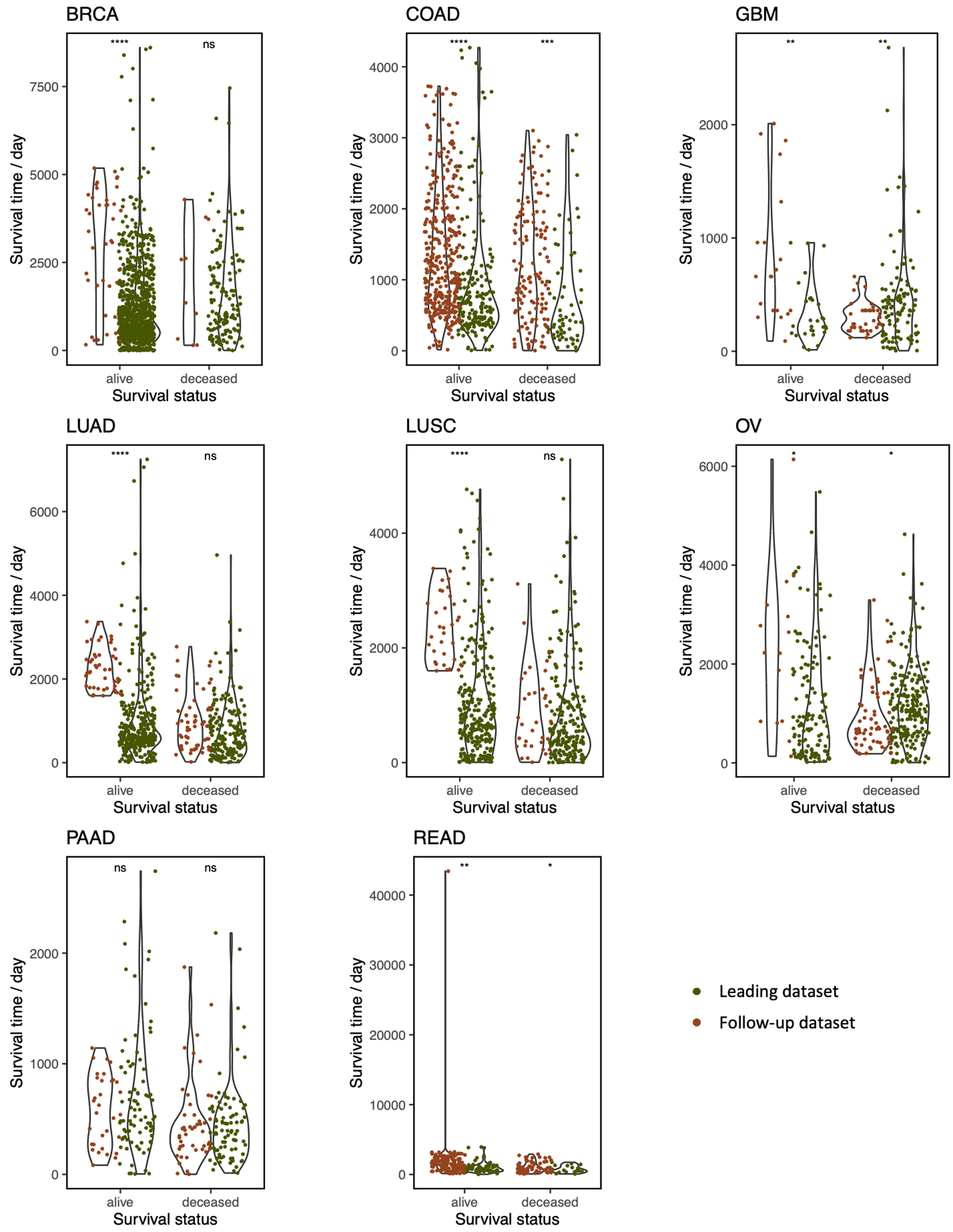

Supplement: Supplementary Fig. S3 — Figure S3. Clinical information overview. [file figs3.jpg]

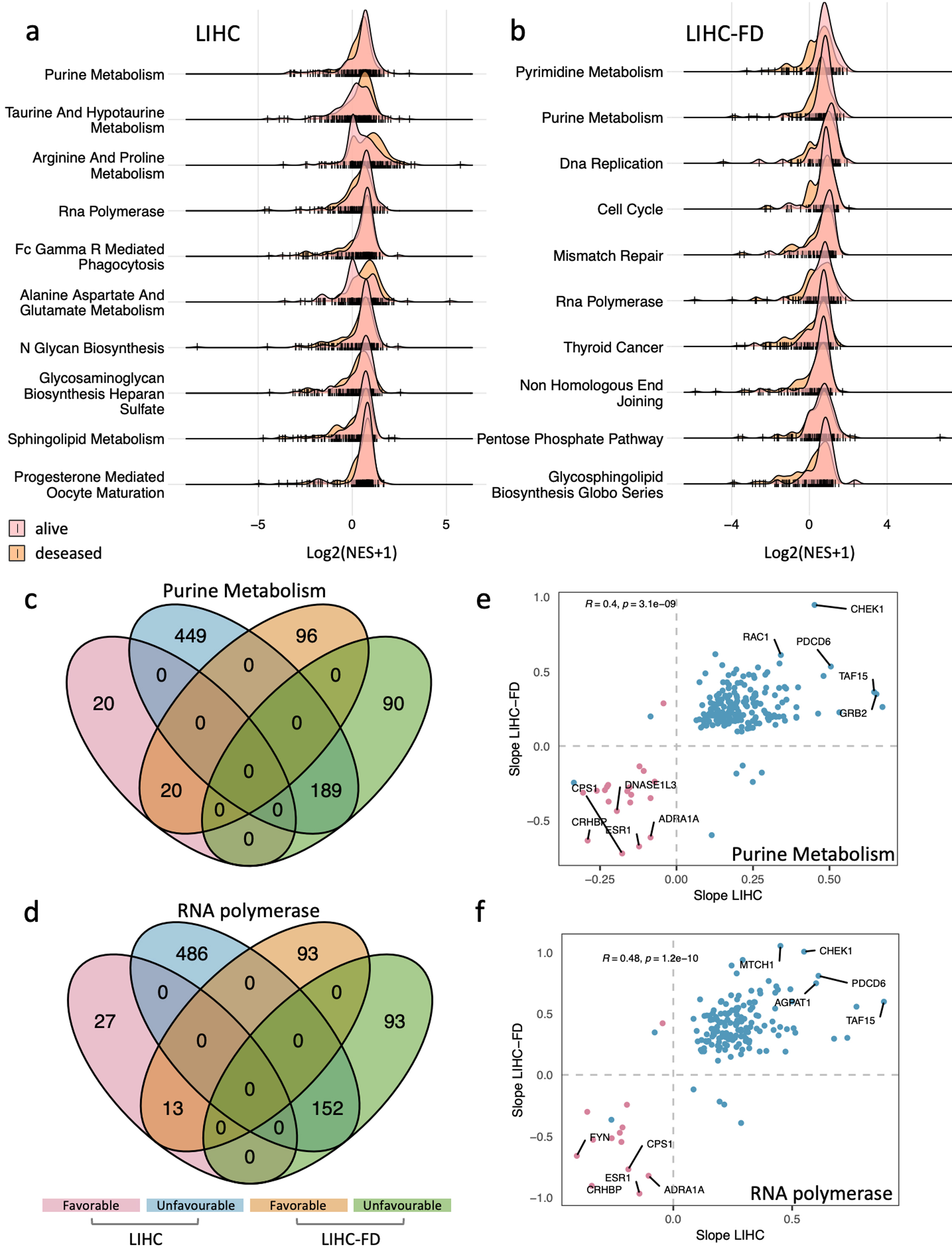

Supplement: Supplementary Fig. S4 — Figure S4. Prognostic pathway regulatory network of LIHC. (a) The pathways that differently activated among alive and deceased patients in LIHC. (b) The pathways that are differently activated among alive and deceased patients in LIHC-FD. (c) The TRs that regulate the purine metabolism pathway are significantly associated with the patient prognosis of LIHC and LIHC-FD. (d) The TRs that regulate the RNA polymerase pathway are significantly associated with the patient prognosis of LIHC and LIHC-FD. (e) The CPGs deprived of (c) and KM analysis showed high consistency of activity in LIHC and LIHC-FD. (f) The CPGs deprived from (d) and KM analysis showed high consistency of activity in LIHC and LIHC-FD. [file figs4.jpg]

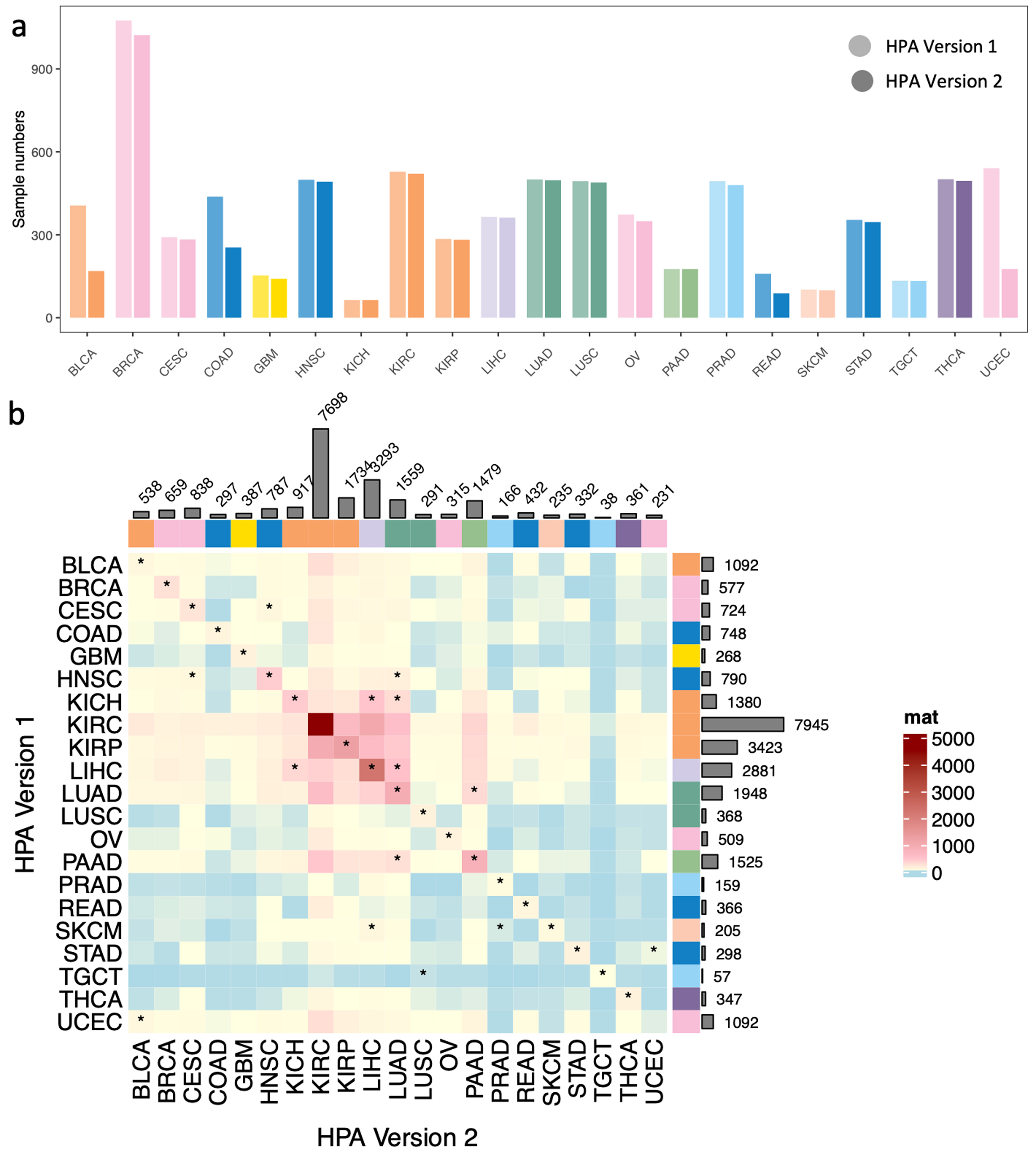

Supplement: Supplementary Fig. S5 — Figure S5. Comparison of two versions. (a) The sample number comparison among 21 cancer types. (b) The Spearman correlation of KM coefficient of prognostic genes among cancer types. The hypergeometric test was applied to examine the overlap significance, p values are denoted as: ∗ p<0.05, ∗∗ p<0.01, and ∗∗∗ p<0.001. [file figs5.jpg]
